# An intraductal papillary neoplasm of the bile duct mimicking a hemorrhagic hepatic cyst: a case report

**DOI:** 10.1186/1477-7819-11-111

**Published:** 2013-05-24

**Authors:** Tatsuhiko Kakisaka, Toshiya Kamiyama, Hideki Yokoo, Kazuaki Nakanishi, Kenji Wakayama, Yosuke Tsuruga, Hirofumi Kamachi, Tomoko Mitsuhashi, Akinobu Taketomi

**Affiliations:** 1 Department of Gastroenterological Surgery I, Hokkaido University Graduate School of Medicine, North 15, West 7, Kita-ku, Sapporo, 060-8638, Japan; 2 Department of Surgical Pathology, Hokkaido University Hospital, North 14, West 5, Kita-ku, Sapporo, 060-8648, Japan

**Keywords:** Hemorrhagic hepatic cyst, Intraductal papillary neoplasm of the bile duct, Oncocytic

## Abstract

An intraductal papillary neoplasm of the bile duct is a biliary, epithelium-lined, cystic lesion that exhibits papillary proliferation and rarely causes large hemorrhagic cystic lesions. Here, we report a case of an intraductal papillary neoplasm of the bile duct mimicking a hemorrhagic hepatic cyst in a middle-aged man with large hemorrhagic hepatic cysts who experienced abdominal pain and repeated episodes of intracystic bleeding. Following portal vein embolization, extended right hepatic lobectomy was performed, and intraoperative cholangiography revealed communication between the intracystic space and the hepatic duct. Although histological studies revealed that the large hemorrhagic lesion was not lined with epithelium, the surrounding multilocular lesions contained biliary-derived epithelial cells that presented as papillary growths without ovarian-like stroma. A diagnosis of oncocytic-type intraductal papillary neoplasm of the bile duct was made, and we hypothesized that intracystic bleeding with denudation of the lining epithelial cells might occur as the cystically dilated bile duct increased in size. Differential diagnosis between a hemorrhagic cyst and a cyst-forming intraductal papillary neoplasm of the bile duct with bleeding is difficult. However, an intraductal papillary neoplasm of the bile duct could manifest as multilocular hemorrhagic lesions; therefore, complete resection should be performed for a better prognosis.

## Background

Intraductal papillary neoplasm of the bile duct (IPNB), which is histologically characterized by the prominent papillary growth of atypical biliary epithelium with distinct fibrovascular cores, is considered to be a biliary counterpart of intraductal papillary mucinous neoplasm of the pancreas [1]. IPNB can develop in the intrahepatic, hilar and extrahepatic regions of the bile duct.

The common clinical signs of IPNB are abdominal pain, jaundice and cholangitis. Radiological imaging studies commonly indicate diffuse bile duct dilatation with or without a papillary mass [2,3]; however, IPNB rarely causes intraductal bleeding followed by the formation of large cystic lesions. Here, we report the surgical treatment of an IPNB mimicking a hemorrhagic hepatic cyst.

## Case presentation

**Figure 1:**
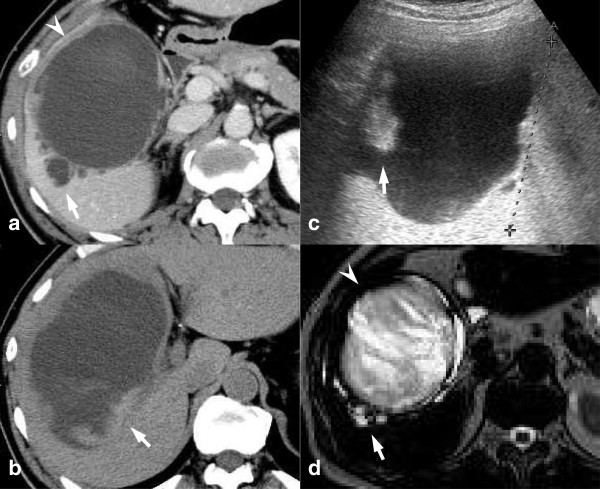
**Imaging study. (a)** Contrast-enhanced computed tomography scan showing the large cystic lesion (*arrowhead*) and surrounding small cystic lesions (arrow). **(b)** Non-enhanced computed tomography scan illustrating high-density components (arrow) in the low-density cystic fluid. **(c)** Abdominal ultrasonography scan showing mural nodules in the large cystic lesion (arrow). **(d)** T2-weighted sequences on magnetic resonance imaging illustrating large (arrowhead) and small cystic lesions (arrow).

A 65-year-old man presented at the hospital with epigastric pain. Laboratory tests indicated mild anemia (hemoglobin, 12.7 g/dl), elevated liver enzymes (aspartate transferase, 69 IU/l; alanine transferase, 131 IU/l), and increased concentrations of total bilirubin (1.4 mg/dl), γ-glutamyl transpeptidase (116 IU/l) and C-reactive protein (5.68 mg/dl). Carcinoembryonic antigen (CEA), carbohydrate antigen 19–9 (CA19-9) and α-fetoprotein were at normal levels. Computed tomography (CT) of the abdomen revealed a large hemorrhagic cyst in the right lobe of the liver. Transarterial embolization of the peripheral portions of the right and middle hepatic arteries was performed for hemostasis. The patient was referred to our hospital and was recommended surgical treatment because additional episodes of intracystic bleeding and abdominal pain were anticipated. Abdominal CT showed a large cystic lesion (13 cm in diameter) with a thickened wall, around which we observed several small cystic lesions (Figure 1a). The large cystic lesion contained low-density fluid with several high-density components (Figure 1b), which were observed as mural nodules on abdominal ultrasonography (Figure 1c). These mural nodules were considered to be intracystic hematomas. Abdominal ultrasonography and magnetic resonance imaging also revealed small multilocular cystic lesions (Figure 1d). On the basis of these findings, a diagnosis of hemorrhagic hepatic cysts was made, and surgical resection was subsequently scheduled. Because the presumptive resected liver volume was 68%, we performed preoperative portal vein embolization to induce hypertrophy of the remnant liver [4].

**Figure 2:**
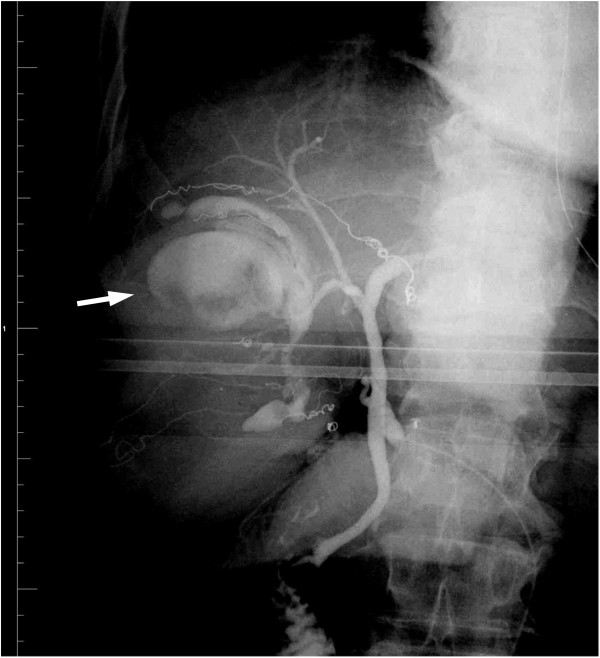
Intraoperative cholangiography illustrating communication between the hepatic duct and the intracystic space (arrow).

The presumptive resected liver volume had decreased to 60% 14 days after the portal vein embolization; the surgical resection was performed on day 18. A laparotomy revealed whitish cystic lesions on the right lobe of the liver and in the inferior region of the left medial section. Intraoperative cholangiography (IOC) is routinely performed before hepatic resection in our institute to map the intrahepatic bile duct [4]. IOC indicated communication between the intracystic space and the hepatic duct (Figure 2), which is a characteristic manifestation of IPNB. We performed an extended right lobectomy and cholecystectomy without reconstruction of the biliary system.

**Figure 3:**
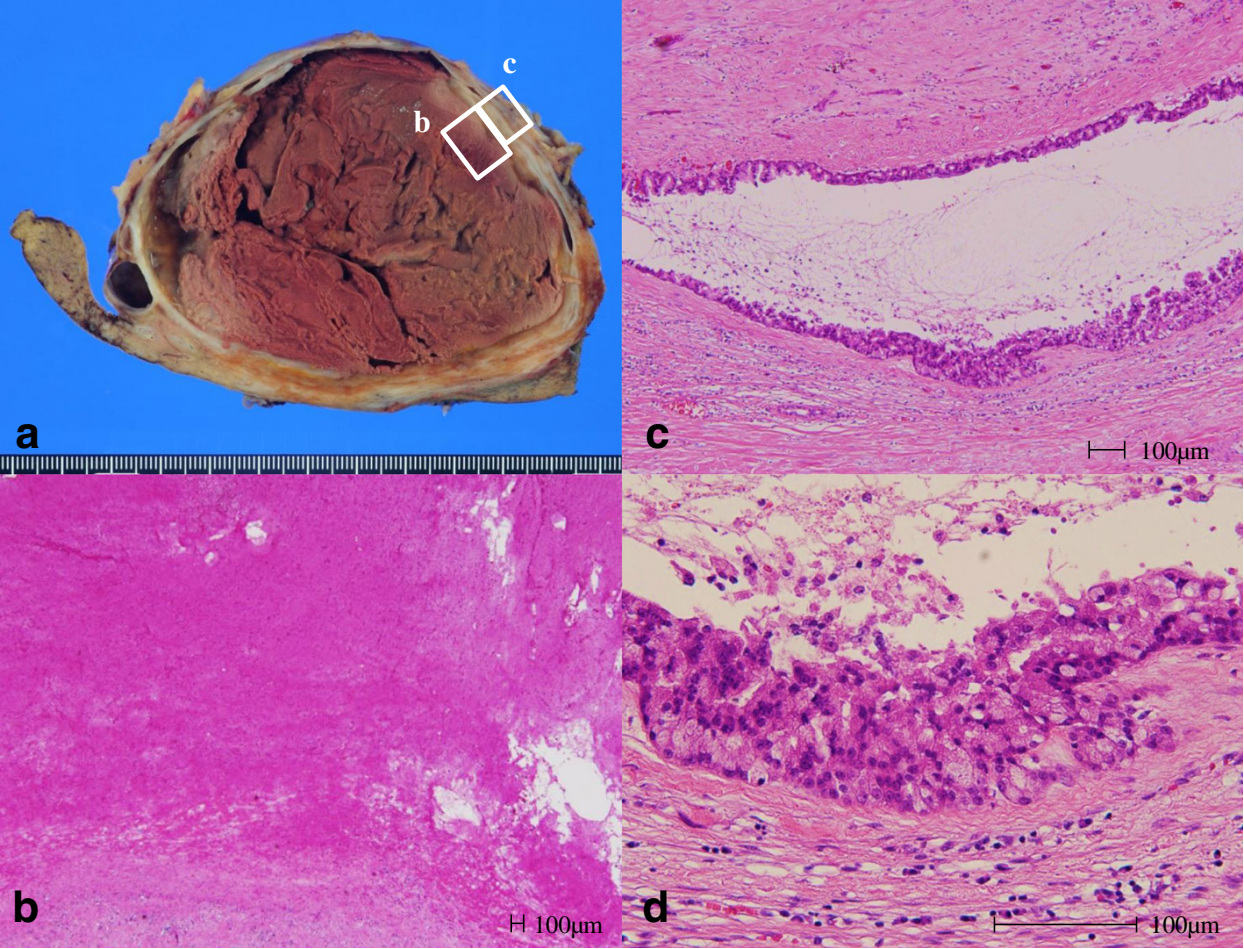
**Gross appearance and histology of the resected section. (a)** The cut surface showing a large cystic lesion with a thickened wall and a hematoma. Boxed areas correspond to the areas shown in **b** and **c**. **(b)** On histology, the large cystic lesion was occupied by a hematoma and not lined with epithelial cells (hematoxylin and eosin stain). **(c)** Small multilocular lesions around the large cystic lesion were lined with epithelial cells (hematoxylin and eosin stain). **(d)** Magnified view of the image in c. These epithelial cells contained abundant eosinophilic cytoplasm and round nuclei, presenting as papillary growths without ovarian-like stroma (hematoxylin and eosin stain).

The large cystic lesion exhibited a white thickened wall and was filled with rust-colored hematoma, as seen in the resected specimen (Figure 3a). The levels of both CEA and CA19-9 were high in the cystic fluid (117.5 ng/ml and 217.5 U/ml, respectively). Histological analysis indicated that the large cystic lesion was not lined with epithelium (Figure 3b); however, the surrounding small multilocular lesions were lined with columnar epithelial cells (Figure 3c). These cells contained abundant eosinophilic cytoplasm and round nuclei, and presented as papillary growths with intermediate-grade neoplastic changes (Figure 3d) and lacked ovarian-like stroma. Immunohistochemical staining indicated that these epithelial cells were positive for cytokeratins 7 and 19, indicating that they were derived from the biliary epithelium. These cells were also positive for mucin core proteins (MUCs) such as MUC5AC and MUC6 with focal expression of MUC1; however, MUC2 expression was weak. Mucin did not accumulate in the cystic lesions. The pathological diagnosis was oncocytic-type IPNB with intermediate-grade intraepithelial neoplasia according to the World Health Organization (WHO) classification system (2010) [5]. Intracystic bleeding with denudation of the epithelial cell lining could have occurred as the cystically dilated bile duct increased in size.

The patient had an uneventful recovery and was discharged 13 days after the operation. At his one-year follow-up visit, the patient was healthy and did not show any signs of recurrence.

## Discussion

Simple hepatic cysts are commonly observed in the general population using various imaging modalities. Although most hepatic cysts are not associated with symptoms, clinical presentations such as pain, biliary obstruction, infection, rupture and compression of other organs infrequently occur. Spontaneous intracystic bleeding is also a rare complication [6-14]. According to the WHO classification system, a hemorrhagic hepatic cyst is defined as a non-neoplastic hepatic lesion without an epithelial lining [5]. Gaviser hypothesized that the epithelium may exhibit necrosis if the intracystic pressure is too high; moreover, desquamated epithelium is associated with injury to surrounding blood vessels, which can cause intracystic bleeding [15].

By contrast, according to the WHO classification system, an intrahepatic IPNB is defined as a cystic lesion that is lined with biliary, mucinous or oncocytic epithelium in papillary configurations without ovarian-like stroma [5]. Luminal communication between the cystic lesions and the bile ducts supports a diagnosis of IPNB. Biliary cystic tumors with bile duct communication could also be regarded as IPNB with prominent cystic dilatation [16]. Biliary cystic changes may occur, followed by twisting or strangulation of the bile duct due to neoplastic overgrowth of IPNB with a blocked bile flow [17]. Mucin overproduction in the affected bile duct can cause biliary cystic changes [16,17], although mucin hypersecretion is observed in only one-third of all IPNB cases [2]. Intraductal bleeding is an uncommon clinical condition in IPNB [2,3]; however, Zen *et al*. observed mucinous fluid associated with hemorrhage in two out of nine cases of cyst-forming IPNBs [16]. Recurrent intracystic hemorrhage, as in this case, may cause rapid enlargement of a small cystic lesion.

A preoperative differential diagnosis between a hemorrhagic hepatic cyst and cyst-forming IPNB with bleeding is difficult when based solely upon clinical and radiological information. Yang *et al*. reported that only 11.5% and 13.5% of patients with an IPNB had high serum levels of CEA and CA19-9, respectively [3]. CEA and CA19-9 levels in the cystic fluid were elevated not only in patients with biliary cystadenoma and cystadenocarcinoma [18,19] but also in patients with simple hepatic cysts [20,21]; therefore, tumor marker levels in serum and cystic fluid are not useful for distinguishing between a hemorrhagic hepatic cyst and cyst-forming IPNB. Radiological imaging studies indicate that a thickened cyst wall or intracystic nodules are present in hemorrhagic hepatic cysts [7-9,11,14,18]. However, most previously reported hemorrhagic hepatic cysts were unilocular, and most cases of cyst-forming IPNB involved multilocular lesions [16], as was the case in our current study. This could represent a characteristic for differential diagnosis. Lim *et al*. reported that some cyst-forming IPNBs might arise from peribiliary glands given their diverticulum-like appearance [22]. Although we detected communication between the intracystic space and the hepatic duct by IOC, endoscopic retrograde cholangiography [16] or magnetic resonance cholangiography may identify this communication preoperatively in suspected cases of cyst-forming IPNBs.

As with an intraductal papillary mucinous neoplasm of the pancreas [23], IPNB is histologically categorized into four types: pancreatobiliary, intestinal, gastric and oncocytic. In IPNB, pancreatobiliary- and intestinal-type tumors are more common, whereas gastric- and oncocytic-type tumors are rare. The intestinal type is more frequently associated with mucin secretion than the pancreatobiliary type [2,3,24]. According to the literature, more than half of all IPNB cases contain carcinoma components, and the pancreatobiliary-type is more commonly associated with invasive carcinoma than the gastric and intestinal types [2,3]. Oncocytic-type IPNB is uncommon, and the postoperative prognosis is not clear.

Surgical therapy is required for IPNB treatment. Kim *et al*. reported that surgically treated patients with pancreatobiliary-type IPNB demonstrated poorer survival than those with the gastric and intestinal types because pancreatobiliary-type IPNB was associated with a higher frequency of invasive carcinoma [2]. A positive surgical margin of the bile duct was associated with poor prognosis [25], but long-term survival may be achieved with complete resection [3]. We performed complete resection of the cyst in this patient, and no recurrence was observed during the follow-up period.

## Conclusions

We report a rare case of IPNB mimicking a hemorrhagic hepatic cyst. Although differential diagnosis between a hemorrhagic cyst and cyst-forming IPNB is difficult, multilocular hemorrhagic lesions could represent IPNB. Because IPNB may harbor carcinoma components, complete resection should be performed for a better prognosis.

## Consent

Written informed consent was obtained from the patient for publication of this case report and any accompanying images. A copy of the written consent is available for review by the Editor-in-Chief of this journal.

## Abbreviations

CA19-9: Carbohydrate antigen 19–9; CEA: Carcinoembryonic antigen; CT: Computed tomography; IOC: Intraoperative cholangiography; IPNB: Intraductal papillary neoplasm of the bile duct; MUC: Mucin core protein; WHO: World Health Organization.

## Competing interests

The authors declare that they have no competing interests.

## Authors’ contributions

All authors contributed equally to this work. All authors read and approved the final manuscript.
